# Prospective, randomized, double-blind, double-dummy, active-controlled, phase 3 clinical trial comparing the safety and efficacy of intranasal dexmedetomidine to oral midazolam as premedication for propofol sedation in pediatric patients undergoing magnetic resonance imaging: the MIDEX MRI trial

**DOI:** 10.1186/s13063-023-07529-0

**Published:** 2023-08-11

**Authors:** Olivia Nzungu Wabelo, Denis Schmartz, Mario Giancursio, Françoise De Pooter, Giulia Caruso, Jean-François Fils, Philippe Van der Linden

**Affiliations:** 1 Department of Anesthesiology, Hôpital Universitaire de Bruxelles – Hôpital universitaire des enfants Reine Fabiola, Brussels, Belgium; 2 Université Libre de Bruxelles, Brussels, Belgium; 3 Department of Anesthesiology, Hôpital Universitaire de Bruxelles – Hôpital Erasme, Brussels, Belgium; 4 Ars Statistica, Nivelles, Belgium; 5 Department of Anesthesiology, Grand Hôpital de Charleroi, Charleroi, Belgium

**Keywords:** MRI, Pediatric sedation, Midazolam, Dexmedetomidine

## Abstract

**Background:**

Children under 6 years who need magnetic resonance imaging usually require sedation to obtain best quality images, but the optimal sedation protocol remains to be determined. In 2018, we showed a 22% interruption in image acquisition during magnetic resonance imaging when performing a propofol-based sedation using a bolus approach. As non-pharmacological premedication is often insufficient to reduce the anxiety of children related to parental separation, pharmacological premedication may be useful to facilitate the induction of anesthesia. In our institution, effective premedication is obtained oral intake of midazolam, though its administration relies on patients’ compliance and could also lead to paradoxical reaction. Dexmedetomidine has a safe profile in the pediatric population and can therefore represent an interesting alternative. The primary objective of this trial is to demonstrate the superiority of intranasal dexmedetomidine compared to oral midazolam as premedication in reducing the occurrence of any event requiring temporary or definitive interruption of the examination to allow anesthesiologist intervention in children undergoing magnetic resonance imaging under propofol sedation.

**Methods:**

In this single-center, prospective, randomized, double-blind, double-dummy, active comparator-controlled, superiority trial, we planned to include 250 patients, aged 6 months to 6 years, undergoing a scheduled magnetic resonance imaging requiring the presence of an anesthesiologist. After informed consent, the patients will be randomized to receive either oral midazolam or intranasal dexmedetomidine as premedication. The data will be analyzed in intention to treat, using Kolmogorov–Smirnov *Z*, chi-square, Wilcoxon, and Mann–Whitney *U* tests. A *P*-value < 0.05 will be considered statistically significant.

**Discussion:**

The MIDEX MRI study will assess the efficacy of intranasal dexmedetomidine compared to oral midazolam to improve the quality of a propofol-based sedation prior to magnetic resonance imaging, without negative repercussion on the postoperative period.

**Trial registration:**

ClinicalTrial.gov NCT05192629. Registered on 14 January 2022. Protocol version 2.1

**Supplementary Information:**

The online version contains supplementary material available at 10.1186/s13063-023-07529-0.

## Administrative information

Note: the numbers in curly brackets in this protocol refer to SPIRIT checklist item numbers. The order of the items has been modified to group similar items (see http://www.equator-network.org/reporting-guidelines/spirit-2013-statement-defining-standard-protocol-items-for-clinical-trials/).

**Table:** 

Title {1}	Prospective, randomized, double-blind, double-dummy, active-controlled, phase 3 clinical trial comparing the safety and efficacy of intranasal dexmedetomidine to oral midazolam as premedication for propofol sedation in pediatric patients undergoing magnetic resonance imaging: the MIDEX MRI trial
Trial registration {2a and 2b}.	ClinicalTrial.gov NCT05192629, registered 14 January 2022.
Protocol version {3}	Protocol version 2.1, dated from 30^th^ of June 2022.
Funding {4}	This protocol is only funded by departmental resources
Author details {5a}	Olivia Nzungu Wabelo, MD: Department of Anesthesiology, *Hôpital Universitaire de Bruxelles – Hôpital universitaire des enfants Reine Fabiola*, Brussels, Belgium; *Université Libre de Bruxelles*, Brussels, Belgium Denis Schmartz, MD: Department of Anesthesiology, *Hôpital Universitaire de Bruxelles – Hôpital Erasme*, Brussels, Belgium; *Université Libre de Bruxelles*, Brussels, Belgium Mario Giancursio, MD: Department of Anesthesiology, *Hôpital Universitaire de Bruxelles – Hôpital universitaire des enfants Reine Fabiola*, Brussels, Belgium Françoise De Pooter, MD: Department of Anesthesiology, *Hôpital Universitaire de Bruxelles – Hôpital universitaire des enfants Reine Fabiola*, Brussels, Belgium Giulia Caruso, MD: Department of Anesthesiology, *Hôpital Universitaire de Bruxelles – Hôpital universitaire des enfants Reine Fabiola*, Brussels, Belgium Jean-François Fils, MSc: Ars Statistica, Nivelles, Belgium Philippe Van der Linden, MD, PhD: Department of Anesthesiology, *Grand Hôpital de Charleroi*, Charleroi, Belgium
Name and contact information for the trial sponsor {5b}	Hôpital Universitaire de Bruxelles, Hôpital Universitaire Des Enfants—Reine Fabiola Av J.J. Crocq, 15 – 1020 Brussels – Belgium Phone: + 3224772945 – Fax: + 3224772399 @: nicolas.deconinck@huderf.be
Role of sponsor {5c}	The sponsor is responsible for the safety of the protocol. He will be informed by the principal investigator of any unanticipated problem, protocol deviation, or any other significant event that arises during the conduct of the study according to protocol specification.

## Introduction

### Background and rationale {6a}

Magnetic resonance imaging (M.R.I.) is a medical procedure that lasts 30 to 45 min, depending on the number of images to be taken and the need for additional images following contrast medium injection. The patient must remain perfectly still during the entire process to obtain best quality images. As children below the age of 6 years are usually not cooperative, these procedures are systematically performed under sedation for this age category in our institution [1].

The optimal sedation protocol for MRI remains to be determined. The choice of sedation agent is based on the experience of the operator, the available resources, and the associated costs [2, 3]. The protocol currently in place at *Hôpital Universitaire des Enfants Reine Fabiola* (H.U.D.E.R.F.; Tertiary Academic Pediatric Hospital, Université libre de Bruxelles) involves iterative intravenous boluses of propofol. Propofol is an ideal sedation agent due to its very short time to action, rapid post-sedation recovery, and high efficacy rate [3, 4].

Preoperative stress, which can be experienced as emotionally traumatic for the child, is associated with agitation on awakening from anesthesia, negative behaviors; and may even extend beyond the perioperative period [5]. Previous experiences of procedural pain and distress can render subsequent contact with healthcare professionals extremely difficult [6]. Chorney et al. reported that more than 40% of children aged 2 to 10 years show some distress during induction of anesthesia [7]. Non-pharmacological means of premedication are often insufficient in the most anxious children [8]. The presence of a parent during the induction of anesthesia, a widespread and systematic practice in our institution, is difficult to achieve in the context of MRI, for practical and safety reasons linked to the magnetic field induced by the MRI device. Moreover, its effectiveness is questioned in the literature [9]. Thus, pharmacological premedication is useful to reduce the anxiety related to parental separation, or even parental stress, and to facilitate the induction of anesthesia [10–12].

At H.U.D.E.R.F., premedication consists of oral midazolam, a benzodiazepine with a short onset of action. It provides sedation, anterograde amnesia, anxiolysis, and a reduction in postoperative vomiting. Although an effective premedication agent [13], it can induce in some instances a paradoxical reaction [14]. Moreover, children’s compliance to midazolam oral intake is quite low because of its unpleasant taste [15].

Dexmedetomidine is a highly selective α-2 agonist with sedative, analgesic and anxiolytic properties, while preserving the upper respiratory tract protective reflexes [3, 15–17], and has a relatively short half-life [18]. According to a report by the Pediatric Sedation Research Consortium (P.S.R.C.), dexmedetomidine has a safe profile and a rate of serious adverse events occurrence of 0.36% in the pediatric population [19]. In addition, intranasal administration of dexmedetomidine is non-invasive, painless, and possesses a high bioavailability [15, 20, 21]. Moreover, its lack of taste and smell [22, 23] does not make it as unpleasant and doesn’t generate a secondary source of stress for the child. Furthermore, Mason et al., among others, demonstrated a synergistic effect of dexmedetomidine with propofol [24, 25].

In 2018, we conducted a retrospective study on the sedation protocol applied in H.U.D.E.R.F. consisting of premedication with oral midazolam and sedation with iterative boluses of propofol. This study concluded that the protocol in place was effective but highlighted a 22% interruption in image acquisition during the procedure, largely due to involuntary movements of the child.

### Objectives {7}

The primary objective is to demonstrate the superiority of intranasal dexmedetomidine compared to oral midazolam as premedication in reducing the occurrence of any event (bradycardia, hypotension, desaturation under 95%, and involuntary movements) requiring temporary or definitive interruption of the examination to allow anesthesiologist intervention in children undergoing MRI under propofol sedation.

Secondary objectives include the analysis of the synergistic effect between dexmedetomidine and propofol, which would be reflected by a lower consumption of propofol when these drugs are associated, compared to the association of midazolam and propofol. We will also evaluate the effect of premedication on upper airway patency when positioning the patient on the MRI table (lower jaw fixation to extend the cervical spine, the use of Guedel cannula, laryngeal mask or orotracheal intubation) and the experience of the anesthesiologist in charge of the patient on the occurrence of any adverse event, as described in the primary objective, requiring the interruption of the MRI. In addition, we will evaluate the impact of dexmedetomidine on the post-sedation period, reflected by the patient’s wake-up time and the length of stay in the recovery room. Furthermore, we will assess the acceptability of intranasal premedication using the CHEOPS scale and the quality of MRI images.

### Trial design {8}

The MIDEX MRI trial is a prospective, randomized, double-blind, double-dummy, active comparator-controlled, superiority trial. The study population is the pre-school child.

## Methods: Participants, interventions and outcomes

### Study setting {9}

Any child aged 6 months to 6 years that need a scheduled MRI in the one-day clinic of the H.U.D.E.R.F. will be screened during the preoperative visit.

### Eligibility criteria {10}

#### Inclusion criteria

- Children of both genders aged 6 months to 6 years
- ASA score I to III
- Requiring standard of care MRI due to the subject’s clinical status, regardless of the underlying pathology
- Sedation carried out by an anesthesiologist

#### Exclusion criteria

- Contraindications to MRI (cardiac pacemaker, neurostimulator, ferromagnetic implant)
- Emergency MRI
- Presence of head trauma
- Presence of nasal congestion or upper respiratory tract infection on the day of sedation
- Multiple procedures during the same sedation (operating room, evoked potentials, etc.)
- Children with pathology requiring airway safety
- Any known allergic or hypersensitivity reaction to dexmedetomidine
- Any known allergic or hypersensitivity reaction to benzodiazepines
- Concomitant use of negative chronotropes, such as Digoxine
- Patient known with chronic respiratory failure or myasthenia
- Patient known with anatomical abnormality of the airway, lung disease, or sleep apnea syndrome
- Patient with known cardiac rhythm abnormality or cardiovascular disease
- Patient with known hepatic disorder or chronic kidney disease
- Patient with hypotension or bradycardia on the day of the examination
- Patient with a BMI > 97th percentile (which corresponds to overweight, including obesity)

### Who will take informed consent? {26a}

The informed consent will be obtained by an anesthesiologist during the preoperative consultation or a member of the unblinded study team on the day of the sedation. Informed consent must be given voluntarily and signed by the legal representative of the patient and an investigator before any study-specific procedure will be initiated.

### Additional consent provisions for collection and use of participant data and biological specimens {26b}

Not applicable; no samples will be collected.

## Interventions

### Explanation for the choice of comparators {6b}

Premedication with intranasal dexmedetomidine is non-invasive, painless, and associated with a good bioavailability [15, 20, 21]. Its lack of taste and smell [22, 23] could lead to a good compliance in the pediatric population. Two micrograms/kilogram is the most common dosage used as premedication according to an international survey among 791 anesthesiologists of the European Society for Pediatric Anesthesiology (E.S.P.A.), Society for Pediatric Anesthesia in New Zealand and Australia (S.P.A.N.Z.A.), Association of Paediatric Anaesthetists of Great Britain and Ireland (A.P.A.G.B.I.) and Society for Pediatric Anesthesia (S.P.A.) [26], and is perfectly safe for the children [27]. Nevertheless, several studies demonstrated the superiority of intranasal dexmedetomidine over oral midazolam, as premedication, in producing satisfactory separation from parents [14, 28–32].

### Intervention description {11a}

On the sedation day, the child will be fasting according to our institutional protocol: the fasting period is 2h for clear liquids (water and fruit juices without pulp), 4h for breast milk, and 6h for infant formula, non-human milk, and solid foods. The medical team will verify that the child is still eligible for the study and that informed consent has been signed by the parents or the legal representative. Child’s state of health will be verified, and vital signs will be recorded before the premedication, as for any anesthesia procedure.

Unblinded authorized site staff (nurse anesthesiologist or research nurse) will prepare the premedication that will be administered by a blinded member of the nursing staff in syringes labeled with the identity of the patient:

- A dose of 0.02 ml/kg of an intranasal solution containing either dexmedetomidine (which corresponds to a dosage of 2 mcg/kg of dexmedetomidine (Dexdor®, 100 mcg/ml) in group dexmedetomidine-propofol) or matching placebo (physiological saline), depending on the group assigned to the patient. This intranasal solution will be administered using a 1-ml syringe with an intranasal Mucosal Atomization Device (Teleflex MAD130®).
- A dose of 0.125 ml/kg of an oral solution containing either midazolam (which corresponds to a dosage of 0.25 mg/kg midazolam (Ozalin®, 2 mg/ml), with a maximum of 20 mg, in group midazolam-propofol) or matching placebo (physiological saline), depending on the group assigned to the patient. This oral solution will be administered using the dedicated applicator, provided by Primex Pharmaceuticals Oy.

A patch of local anesthetic (Emla® or Rapydan®) will be placed on the skin to reduce the pain associated with the insertion of an intravenous (I.V.) catheter, which will be necessary for the administration of propofol. At the same time, the patient will receive the premedication following the double-dummy technique. After the administration of the premedication, vital signs will be recorded.

After 30 min, the child will be taken to the induction room, located next to the MRI room, and an intravenous catheter will be inserted by the anesthesiologist in the previously anesthetized skin area. The sedation will be carried out by this anesthesiologist, blinded to the premedication received by the child, using propofol. A propofol I.V. bolus of 2 to 6 mg/kg will be administered until deep sedation (i.e., a University of Michigan Sedation Scale score greater than or equal to 3) [33]. An additional bolus of 0.5 to 1 mg/kg may be given if necessary, depending on the child's tolerance and the duration of the MRI (which is usually longer if an injection of contrast medium is required).

The usual MRI-compatible monitoring (Philips Expression MR400®) that will be used includes an electrocardiogram, a pulse oximeter, and a capnograph. The respiratory rate will be calculated from the capnography trace. It will be completed by measuring non-invasive blood pressure at the reception of the child in the 1-day clinic (reference measurement), when the patient will be placed on the MRI table, and at the end of the examination (to avoid any stimuli that could cause a reaction movement). The child’s neck will be held in hyperextension using a roller under the shoulder and an oxygen mask applied to the child's face, under which the capnograph probe will be slipped. If necessary, the anesthesiologist may fix the inferior jaw to maintain the cervical spine in hyperextension, use a Guedel cannula, a laryngeal mask, or an endotracheal tube.

After the sedation, the child will be monitored in the post-anesthesia care unit (P.A.C.U.). During the entire awakening period, parameters such as oxygen saturation, breathing rate, blood pressure and heart rate will be recorded on a monitoring sheet. Moreover, the patient will be assessed every 10 min, using Aldrete and Chung scores. The Aldrete score determines when the child can start consuming clear drinks, as well as the withdrawal of the infusion if the drink is well tolerated. The Chung score determines when the patient can leave the monitoring room. Monitoring will be stopped as soon as a score greater than or equal to 9 (out of 10) is obtained using the two scores. In addition, the times of arrival in the recovery room, and time of discharge will be recorded.

Two radiologists will evaluate the quality of MRI images based on the presence of motion-related artifacts. The examinations will be classified as “no artifact” or “with artifact.” The second category (“with artifacts”) will be assessed for the occurrence of motion artifacts based on the full duration of the examinations. Radiologists will not be aware of the type of premedication that has been administered to the patient.

All data will be collected on the case report form (REDCap database).

### Criteria for discontinuing or modifying allocated interventions {11b}

Not applicable. This trial consists in a single administration of midazolam or dexmedetomidine following the randomization.

### Strategies to improve adherence to interventions {11c}

Not applicable. This trial consists in a single administration of midazolam or dexmedetomidine following the randomization.

### Relevant concomitant care permitted or prohibited during the trial {11d}

No relevant concomitant care will be prohibited during the trial. Patient taking negative chronotropes are excluded from the trial (cf. exclusion criteria).

### Provisions for post-trial care {30}

Not applicable. Both treatments are standard of care, and no follow-up is needed.

### Outcomes {12}

#### Primary outcome measure

Incidence and type of any event occurring during the MRI procedure requiring temporary or definitive interruption of the examination to allow anesthesiologist intervention:

- Bradycardia, defined as a decrease of 2 standard deviations below normal for age, as described by the American Heart Association (AHA) in the Pediatric Advanced Life Support (PALS) manual, and which requires intervention by the anesthesiologist in charge of the patient to improve heart rate and cardiac output [34]
- Hypotension, defined as a systolic blood pressure below the 5.th percentile for age, as described by the AHA in the PALS manual, and which requires intervention by the anesthesiologist in charge of the patient to improve blood pressure [34]
- Desaturation under 95%, defined as moderate if SpO2 is below 95%, and severe if below 90% [34]
- Involuntary movements before the end of the examination

#### Secondary outcome measures

- Cumulative dose in mg/kg of propofol administered during the procedure
- Need of additional bolus of propofol and total dose administered in mg
- Time to Aldrete score > 9/10 and Chung score > 9/10
- Length of stay in the post-anesthesia care unit in minutes
- Upper airway patency when positioning the patient on the MRI table: fixation of inferior jaw to extend the cervical spine, Guedel cannula, laryngeal mask, endotracheal tube
- Experience of the anesthesiologist in charge of the patient
- Premedication’s acceptability using the CHEOPS scale
- Classification of MRI images by two radiologists into 2 groups: “without artifact” and “with artifact”

### Participant timeline {13}

#### Sample size {14}

In our 2018 database, we have reported an incidence of 22% of events that interrupted the examination in the population aged 0 to 6 years (*N* = 90). To observe a reduction in this incidence by 50% with the association dexmedetomidine-propofol compared to the association midazolam-propofol, with a power of 0.8 and a α of 0.05, a sample of 112 patients per group is required. Considering a 10% drop-out rate, a sample size of 125 patients per group will be appropriate. Based on our activity in MRI sedation, an enrollment time of 12 months is expected.

#### Recruitment {15}

Any child aged 6 months to 6 years who will need a scheduled MRI in the one-day clinic of our institution will be screened during the preoperative visit. During the consultation, the anesthesiologist will explain the MIDEX MRI trial and answer to the questions of the parents or the legal representative if needed. The informed consent will be obtained by the anesthesiologist during the preoperative consultation or a member of the unblinded study team on the day of the sedation.

## Assignment of interventions: allocation

### Sequence generation {16a}

Each eligible patient will be randomized to a premedication group according to a randomization plan in a 1:1 ratio, using a validated randomization software (https://www.sealedenvelope.com). The randomization block size is left to the discretion of the clinical research unit.

### Concealment mechanism {16b}

The randomization will be done using sequentially numbered, opaque, and sealed envelopes. A list containing the patient's identification number and his rank will be kept by the clinical research unit to guarantee the confidentiality of the data.

Investigators and site staff performing the sedation and the assessments will remain blinded to the identity of the investigational treatment from the time of randomization until the database unlock for the data analysis.

### Implementation {16c}

The randomization will be done by a member of the clinical research unit who prepares the randomization envelopes. The anesthesia team will recruit the patients during the preoperative consultation.

## Assignment of interventions: blinding

### Who will be blinded {17a}

The members of the unblinded study team (nurse anesthesiologist or research nurse) will be responsible for the compounding of premedication for study participants. Investigators and site staff performing the sedation and assessments during the pre-sedation and the post-sedation periods will be blinded: the premedication will be administered by a blinded member of the nursing staff who takes charge of the patient in the one-day clinic. The sedation will be carried out by an anesthesiologist, who will be blinded to the premedication received by the child.

The patient will also be blinded, using the double dummy technique.

### Procedure for unblinding if needed {17b}

Unblinding will occur in the case of participant emergencies and after the data analysis. It is the principal investigator’s responsibility to maintain blinding at the clinic site.

If needed to ensure adequate treatment where the principal investigator deems identification of the study drug necessary, the principal investigator or delegate can perform unblinding. The clinical research unit, having a list of patients containing their patient number, rank, and assigned group, may provide emergency disclosure of unblinding information to the Investigator. In this case, the circumstances surrounding the breaking of the blinding code will require documentation. The Investigator should determine and document “causality” prior to unblinding the study drug.

This assignment will be revealed to the principal investigator and the study team after the statistical tests have been carried out and the results obtained.

## Data collection and management

### Plans for assessment and collection of outcomes {18a}

A data collection sheet (the case report form) will be placed at the disposal of the entire study team to group all the needed data in a single document during the exam day. Follow-up will consist of a phone call to the parents or the legal representative the day after the exam.

### Plans to promote participant retention and complete follow-up {18b}

This trial consists in a single administration of midazolam or dexmedetomidine following the randomization. Patients will be monitored during the day of the sedation until discharge from the PACU. All study patients, except those that withdraw consent for the study, will be called 24 h after the sedation to complete the assessment of the post-sedation period.

### Data management {19}

The principal investigator is responsible for ensuring the accuracy, completeness, legibility, and timeliness of the data reported. The investigators will maintain adequate case histories of study participants, including accurate case report forms and source documentation. The study case report form is the data collection instrument for the study. Study data will be recorded with the electronic case report forms named REDCap (Vanderbilt University, USA), on the intrahospital server of H.U.D.E.R.F.

### Confidentiality {27}

All patient data collected will be managed by the sponsor to ensure the confidentiality of those data, and in accordance with applicable national laws and regulations on personal data protection, and the study will perform in accordance with WHO guidelines for Good Clinical Practice. The case report form will be stored safely, and the study data will be recorded with REDCap, on the intrahospital server at H.U.D.E.R.F.

### Plans for collection, laboratory evaluation, and storage of biological specimens for genetic or molecular analysis in this trial/future use {33}

Not applicable. There is no biological specimen or molecular or genetic analysis in our study.

## Statistical methods

### Statistical methods for primary and secondary outcomes {20a}

The analyses will be done in intention to treat. Data will be analyzed for normality using the Kolmogorov–Smirnov *Z* test. Demographic data will be presented as mean and standard deviation and will be analyzed using the chi-square test. Data not normally distributed will be presented as median and interquartile range and will be analyzed by a Wilcoxon rank test. Ordinal data will be analyzed using the Mann–Whitney *U* test.

A *P*-value < 0,05 will be considered as statistically significant.

### Interim analyses {21b}

Not applicable. No interim analyses are planned.

### Methods for additional analyses (e.g., subgroup analyses) {20b}

The patients will be divided in two groups: midazolam-propofol and dexmedetomidine-propofol, following the randomization. There are no planned subgroup analyses.

### Methods in analysis to handle protocol non-adherence and any statistical methods to handle missing data {20c}

The analyses will be done in intention to treat: all subjects will be analyzed according to the block they have been randomized to. Moreover, this trial consists in a single administration of midazolam or dexmedetomidine, a monitoring until validation of discharge criteria (Aldrete and Chung scores), and a 24-h follow-up by phone consultation. The amount of missing data is expected to be small.

The characteristics of patients who have missing data and the reason will be reported.

### Plans to give access to the full protocol, participant-level data and statistical code {31c}

Access to the protocol and the results will be granted on reasonable request to the authors.

## Oversight and monitoring

### Composition of the coordinating center and trial steering committee {5d}

This investigator-initiated trial will be coordinated by the Department of Anesthesiology of the H.U.D.E.R.F. The evaluation of the study progress will be done monthly.

### Composition of the data monitoring committee, its role and reporting structure {21a}

No special data monitoring is needed for this single-center study using 2 standard of care treatments.

### Adverse event reporting and harms {22}

Adverse events of special interest and serious adverse events, whether related or unrelated, will be reported to the sponsor within 24 h using the appropriate expedited report form. Other supporting documentation of the event may be requested by the sponsor and should be provided as soon as possible. New information regarding the adverse events of special interest or serious adverse events, if not mentioned in the first report, will be sent to the sponsor by the principal investigator. This report will be submitted spontaneously as soon as new elements are known.

A serious adverse event considered completely unrelated to another previously reported serious adverse event will be reported separately as a new incident.

Serious adverse event reports will be archived in the Investigator Site File.

The sponsor will be responsible for notifying the regulatory authority and ethics committee of any unexpected fatal or life-threatening suspected adverse reaction as soon as possible but in no case later than 7 calendar days after the sponsor's initial receipt of the information. The sponsor will notify the regulatory authority and ethics committee of any other suspected unexpected serious adverse reaction as soon as possible but in no case later than 15 calendar days after the sponsor's initial receipt of the information.

### Frequency and plans for auditing trial conduct {23}

The trial will be audited by the Belgian authorities and the ethics committee on a yearly basis.

### Plans for communicating important protocol amendments to relevant parties (e.g., trial participants, ethical committees) {25}

For any change in the protocol, we will contact the ethics committee for approval. Patients will have access to the results of the study.

### Dissemination plans {31a}

After study completion and finalization of the study report, the results of this study will be submitted for publication in a peer-reviewed journal.

## Discussion

Dexmedetomidine is an interesting alternative to midazolam as premedication and could result in a good adherence of young patients. Several studies demonstrated the superiority of dexmedetomidine over midazolam in terms of producing satisfactory separation from parents when used as premedication [14, 28–32].

Neither of these studies investigated the use of dexmedetomidine as premedication for short ambulatory procedures as MRI (less than an hour), and its potential effects on the post-sedation period by comparing the time to recovery and the length of stay in the hospital before discharge after the administration of midazolam or dexmedetomidine.

In addition, the use of dexmedetomidine could optimize a propofol-based sedation by synergistic effect with propofol [25, 26], ensure better quality MRI images and decrease the risk of repeated MRI due to movements artifacts or interrupted examination by an event requiring the intervention of anesthesiologists.

Moreover, increasing numbers of clinical research have reported that propofol may induce developmental neurotoxicity in young patients and infants. The synergistic effect between propofol and dexmedetomidine would result in a decrease in the total dose of propofol administered during sedation. Even if the literature does not indicate if this effect is dose dependent, in vitro experimental models indicate that dexmedetomidine may have a neuroprotective effect on propofol-induced neurotoxicity; the underlying mechanisms have not yet been fully understood [35].

### Trial status

The study (protocol version 2.1) started to enroll patients in October 2022. The recruitment will approximately be completed in September 2023.

### Supplementary Information

**Additional File 1.** Spirit Checklist.

## Data Availability

The MIDEX MRI steering committee including the trial statistician has full access to the trial data. Data will also be available for site trial-related monitoring, audits, Independent Ethics Committee or Institutional Review Board review, and regulatory inspection(s). Any data required to support the protocol can be supplied on request.

## Abbreviations

H.U.D.E.R.F.: Hôpital Universitaire des Enfants Reine Fabiola
I.V.: Intravenous
M.R.I.: Magnetic resonance imaging
P.A.C.U.: Post-anesthesia care unit
